# Highly-expressed lncRNA FOXD2-AS1 in adipose mesenchymal stem cell derived exosomes affects HaCaT cells via regulating miR-185-5p/ROCK2 axis

**DOI:** 10.1080/21623945.2023.2173513

**Published:** 2023-02-12

**Authors:** Huanchao Chang, Junliang Chen, Kun Ding, Tianling Cheng, Shengjian Tang

**Affiliations:** a Plastic Surgery of Plastic Surgery Hospital, Weifang Medical University, Weifang, China; b Vascular surgery department, Affiliated Hospital of Weifang Medical College, Weifang, China; c Burn plastic surgery, The First Affiliated Hospital of Xi’an Medical University, Xi’an, China

**Keywords:** Exosomes, FOXD2-AS1, wound healing, miR-185-5p, ROCK2

## Abstract

The healing of skin wounds is a highly coordinated multi-step process that occurs after trauma including surgical incisions, thermal burns, and chronic ulcers. In this study, the authors investigated lncRNA FOXD2-AS1 function in adipose mesenchymal exosomes from ADMSCs that were successfully extracted. Highly expressed lncRNA FOXD2-AS1 in ADMSCs-exosomes accelerated HaCaT cell migration and proliferation. LncRNA FOXD2-AS1 negatively targeted miR-185-5p, and miR-185-5p negatively targeted ROCK2. Highly expressed lncRNA FOXD2-AS1 in ADMSCs-exosomes promoted HaCaT cell migration and proliferation via down-regulating miR-185-5p and further up-regulating ROCK2. In conclusion, LncRNA FOXD2-AS1 overexpression in ADMSCs derived exosomes might accelerate HaCaT cell migration and proliferation via modulating the miR-185-5p/ROCK2 axis.

## Introduction

The healing of skin wounds is a highly coordinated multi-step process that occurs after trauma, including surgical incisions, thermal burns, and chronic ulcers [1]. The failure of proceeding through such orderly and timely reparation can induce chronic non-healing wounds including diabetic, venous, and decubitus skin ulcers [2,3]. Refractory wound is a huge burden to both the patient and society. Thus, exploring a novel marker for wound healing therapy and clarifying the mechanisms of this fatal disease are imperative.

Mesenchymal stem cells (MSCs) are one type of multipotent progenitor cells that are derived from bone marrow, umbilical cord, and adipose tissue and play a well-known function in tissue regeneration [4]. In recent years, more and more studies have strongly proved that exosomes derived from adipose-derived mesenchymal stem cells (ADMSCs) are safe and have become the hot pot of many researches in many different fields, such as wound healing [5–7]. A previous study has demonstrated that ADMSCs-derived exosomes can accelerate cell proliferation and migration through regulating Wnt/β‐catenin pathway in cutaneous wound healing [8]. ADMSCs-derived exosomes are reported to promote wound healing via accelerating keratinocyte migration and proliferation [9]. Moreover, human ADMSCs are an attractive resource for wound healing due to their regenerative ability to promote injury repair [10]. A study has confirmed that ADMSCs could accelerate wound healing through optimizing fibroblasts characteristics [11], which further verified their regenerative ability to promote injury repair in wound healing.

Long noncoding RNAs (lncRNAs) have been reported to exist in exosomes and modulate gene expression in host cells through intercellular communication [12]. For example, the high expression of lncRNA H19 in ADMSCs-exosomes can up-regulate the expression of SOX9 through miR-19b to promote wound healing [13]. A study has proven that exosomal lncRNA FOXD2-AS1 can act as the promising biomarkers for the diagnostics of colorectal cancer [14]. LncRNA FOXD2-AS1 promotes the progression of a variety of tumours [15–18]. Moreover, lncRNA FOXD2-AS1 promotes the proliferation of many kinds of cells, including trophoblast cell [19], fibroblast-like synoviocytes [20], and chondrocyte [21]. However, the evidence regarding the implication of exosomal lncRNA FOXD2-AS1 derived from ADMSCs in wound healing is lacking.

In the current study, we paid attention to evaluate the effect of exosomal lncRNA FOXD2-AS1 derived from ADMSCs in wound healing and its potential mechanisms. Our findings demonstrated that highly expressed lncRNA FOXD2-AS1 in ADMSCs derived exosomes accelerated HaCaT cell migration and proliferation via the regulation of miR-185-5p/rho-associated coiled-coil-containing protein kinase 2 (ROCK2) axis, suggesting that exosomal lncRNA FOXD2-AS1 derived from ADMSCs may be a novel therapeutic strategy for wound healing.

## Results

### Identification of ADMSCs and exosomes

**Figure 1.**
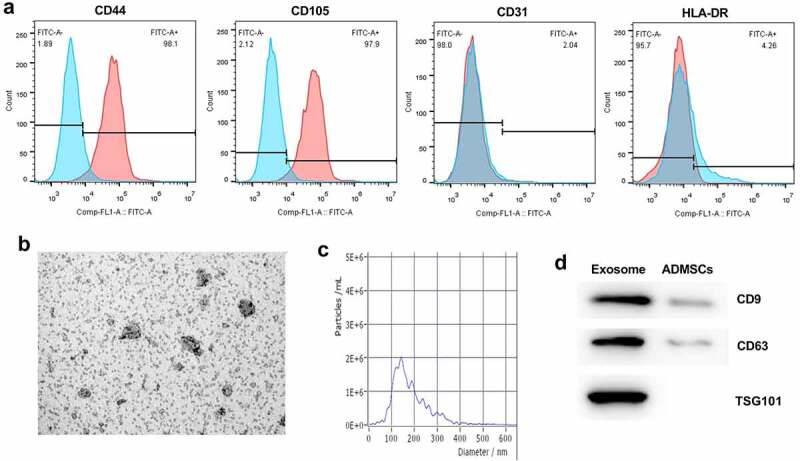
Identification of ADMSCs and exosomes. (a) Flow cytometric analysis of cell surface protein expression of isolated ADMSCs. (b) The ultrastructure of ADSCs-exo under TEM. (c) Exosomes size distribution was analysed using NTA. (d) The expression of CD9, CD63 and TSG101 was detected using western blot.

Flow cytometric analysis was utilized to examine the cell surface protein expression of isolated ADMSCs. As shown in Figure 1a, ADMSCs were positive with CD44 and CD105, but negative with CD31 and HLA-DR. Then, we further identified ADMSCs-exomoses through TEM (Figure 1b). Furthermore, the data of NTA confirmed that the particle size of the extracted exosomes was mainly distributed at about 100 nm (Figure 1c). Moreover, the presentation of specific biomarkers CD9, CD63 and TSG101 indicated that the exosomes were successfully extracted (Figurerue 1D).

### Highly expressed lncRNA FOXD2-AS1 in ADMSCs-exosomes accelerated HaCaT cell migration and proliferation

**Figure 2.**
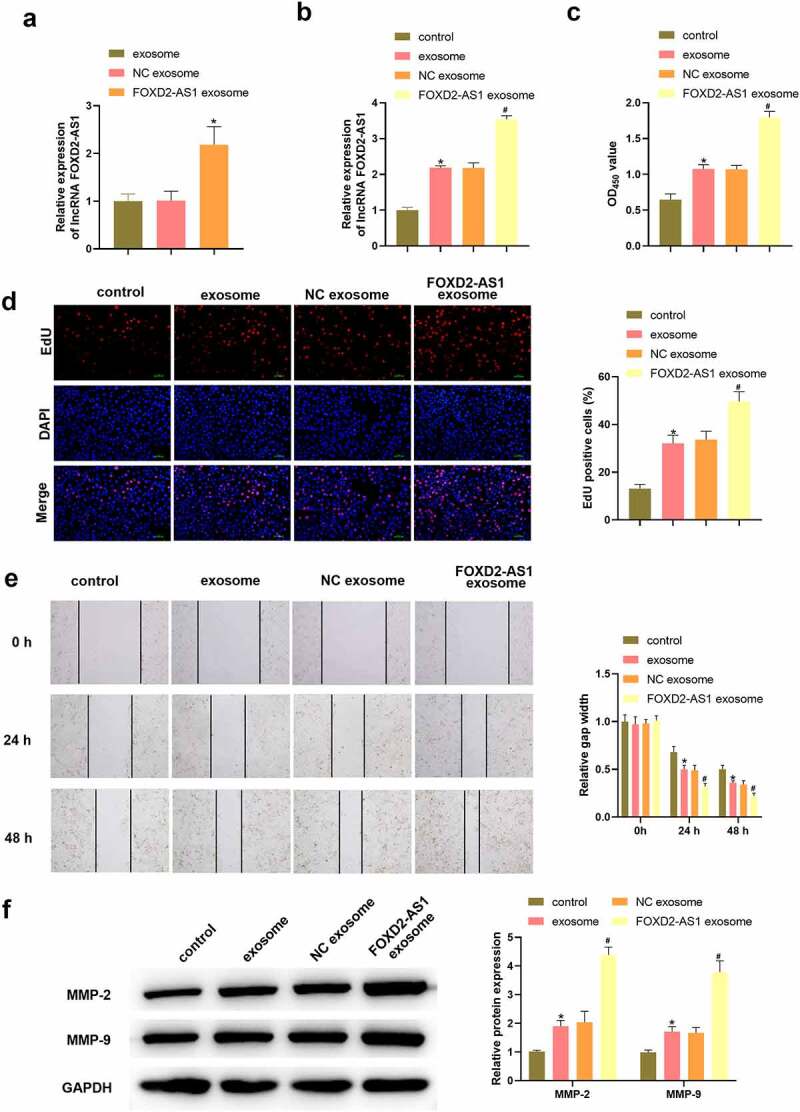
Highly expressed lncRNA FOXD2-AS1 in ADMSCs-exosomes promoted the migration and proliferation of HaCaT cells. Following different treatment, the expression of lncRNA FOXD2-AS1 in ADMSCs-exosomes was detected using qRT-PCR (a); the expression of lncRNA FOXD2-AS1 in HaCaT cells was detected using qRT-PCR (b); HaCaT cell variety was evaluated using CCK-8 assay (B); HaCaT cell proliferation was determined with EdU assay (c); HaCaT cell migration at 0, 24, 48 h was analysed with wound healing assay (d); MMP-2 and MMP-9 level were tested utilizing western blot (e). *P < 0.05 vs. exosome or control group; ^#^P < 0.05 vs. NC exosome group.

We overexpressed lncRNA FOXD2-AS1 in ADSCs and extracted exosomes from ADSCs. The level of lncRNA FOXD2-AS1 was significantly upregulated following lncRNA FOXD2-AS1 overexpression plasmid transfection (Figure 2a). As shown in Figure 2b, treatment with exosomes significantly increased lncRNA FOXD2-AS1 expression in HaCaT cells, and lncRNA FOXD2-AS1 overexpression-transfected exosomes further elevated lncRNA FOXD2-AS1 expression. CCK-8 and EdU assays data proved that exosomes treatment markedly promoted the proliferation of HaCaT cells (Figure 2c and 2d). Meanwhile, the proliferation was significantly increased in FOXD2-AS1 exosome group relative to NC exosome group (Figure 2c and 2d). In addition, we assessed the migrative function of highly expressed lncRNA FOXD2-AS1 in exosomes by wound healing analyses. Figure 2e confirmed that exosomes treatment markedly increased HaCaT cell migration, and lncRNA FOXD2-AS1 overexpression-transfected exosomes further elevated HaCaT cell migration compared with NC exosomes. Similarly, the results are also demonstrated using western blot by detecting MMP-2 and MMP-9 (figure 2f).

### Knockdown lncRNA FOXD2-AS1 in ADMSCs-exosomes inhibited HaCaT cell migration and proliferation

**Figure 3.**
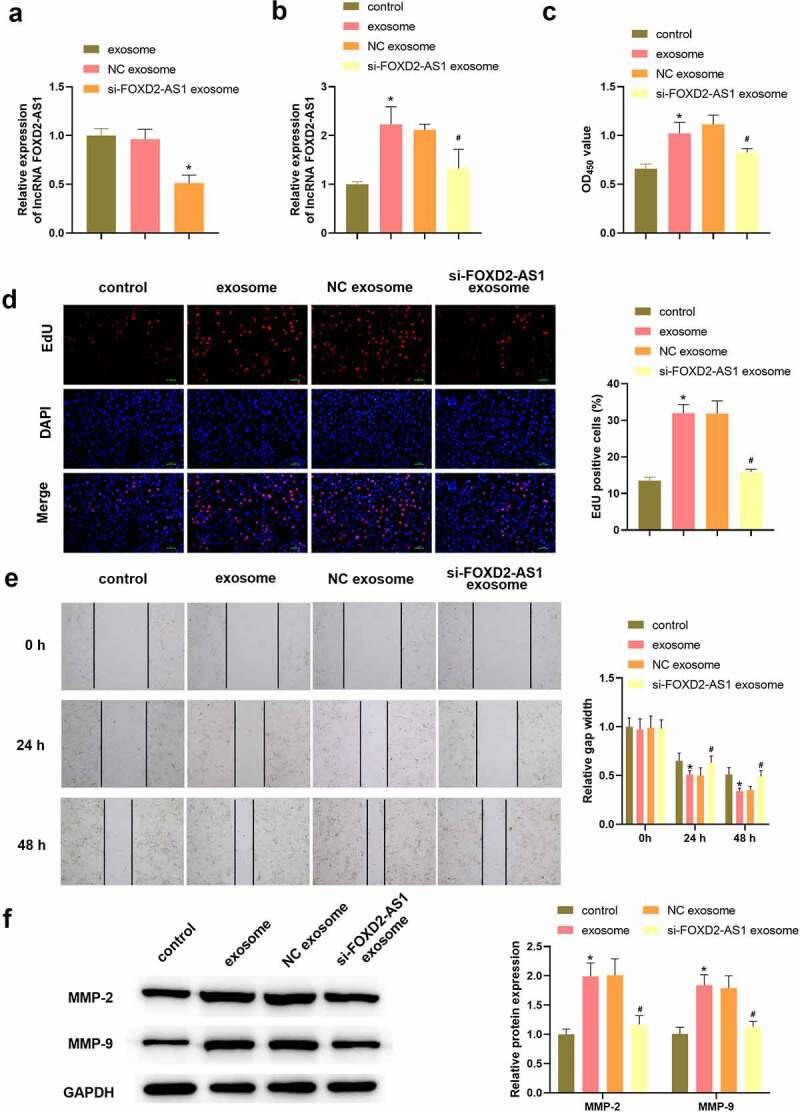
Knockdown lncRNA FOXD2-AS1 in ADMSCs-exosomes inhibited HaCaT cell migration and proliferation. Following different treatment, the expression of lncRNA FOXD2-AS1 in ADMSCs-exosomes was detected using qRT-PCR (a); the expression of lncRNA FOXD2-AS1 in HaCaT cells was detected using qRT-PCR (b); HaCaT cell variety was evaluated using CCK-8 assay (B); HaCaT cell proliferation was determined with EdU assay (c); HaCaT cell migration at 0, 24, 48 h was analysed with wound healing assay (d); MMP-2 and MMP-9 level were tested utilizing western blot (e). *P < 0.05 vs. exosome or control group; ^#^P < 0.05 vs. NC exosome group.

We knockdown lncRNA FOXD2-AS1 in ADSCs and extracted exosomes from ADSCs. The level of lncRNA FOXD2-AS1 was significantly downregulated following si-lncRNA FOXD2-AS1 transfection (Figure 3a). Exosomes significantly increased lncRNA FOXD2-AS1 expression in HaCaT cells, and si-lncRNA FOXD2-AS1 transfection exosomes decreased lncRNA FOXD2-AS1 expression (Figure 3b). CCK-8 and EdU assays data proved that the proliferation was significantly decreased in si-FOXD2-AS1 exosome group relative to the NC exosome group (Figure 3c and 3d). si-FOXD2-AS1 transfected exosomes decreased HaCaT cell migration (Figure 3e). Similarly, the results are also demonstrated using western blot by detecting MMP-2 and MMP-9 (figure 3f).

### lncRNA FOXD2-AS1 negatively targeted miR-185-5p

**Figure 4.**
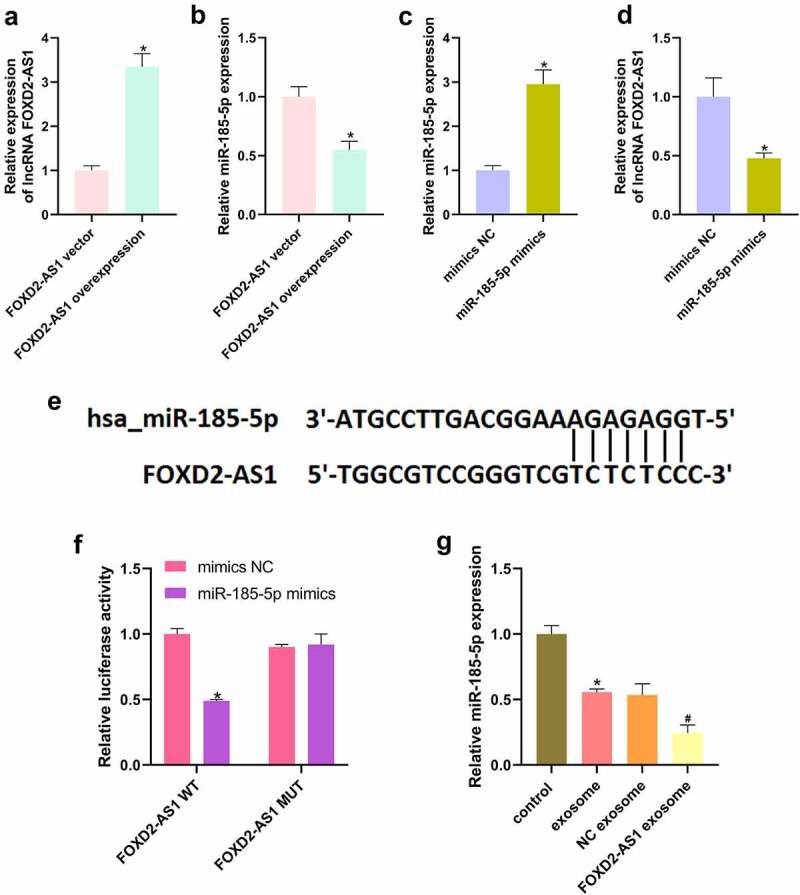
lncRNA FOXD2-AS1 negatively targeted miR-185-5p. After transfection of lncRNA FOXD2-AS1 overexpression plasmid, lncRNA FOXD2-AS1 (a) and miR-185-5p (b) levels were tested using qRT-PCR. Following transfection of miR-185-5p mimics, the expression of miR-185-5p (c) and lncRNA FOXD2-AS1 (d) was tested using qRT-PCR. (e) The bind sites of lncRNA FOXD2-AS1 and miR-185-5p. (f) A dual luciferase reporter assay verified the binding relationship between lncRNA FOXD2-AS1 and miR-185-5p. (D) After treatment of exosomes and lncRNA FOXD2-AS1 overexpression-transfected exosomes, qRT-PCR was utilized to assess miR-185-5p level (g). *P < 0.05 vs. FOXD2-AS1 vector, mimics NC, mmics NC + FOXD2-AS1 WT group, and control group, ^#^P < 0.05 vs. NC exosome group.

After transfection of lncRNA FOXD2-AS1 overexpression vector in HaCaT cells, lncRNA FOXD2-AS1 level was significantly upregulated (Figure 4a), and the expression of miR-185-5p was markedly decreased (Figure 4b). Additionally, following transfected with miR-185-5p mimics in HaCaT cells, miR-185-5p level was elevated (Figure 4c) and lncRNA FOXD2-AS1 level was reduced (Figure 4d). The predicted binding sites between lncRNA FOXD2-AS1 and miR-185-5p are shown in Figure 3e. Furthermore, miR-185-5p mimics notably inhibited luciferase activities in FOXD2-AS1-WT group, while had no function on luciferase activities in FOXD2-AS1-MUT group (Figure 4f). Moreover, we also found that exosomes treatment only significantly reduced miR-185-5p level, and lncRNA FOXD2-AS1 overexpression-transfected exosomes further inhibited the expression of miR-185-5p (Figure 4g).

### Highly expressed lncRNA FOXD2-AS1 in ADMSCs-exosomes promoted HaCaT cell migration and proliferation via down-regulating miR-185-5p

**Figure 5.**
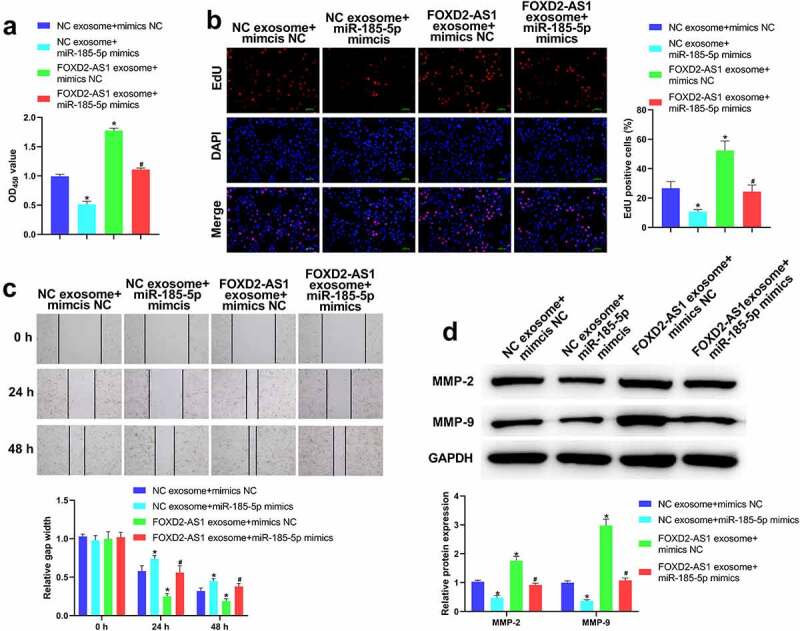
Highly expressed lncRNA FOXD2-AS1 in ADMSCs-exosomes promoted HaCaT cell migration and proliferation via down-regulating miR-185-5p. Following different treatment, the HaCaT cell variety was assessed utilizing CCK-8 analyses (a); HaCaT cell proliferation was evaluated applying EdU analyses (b); HaCaT cell migration at 0, 24, 48 h was analysed with wound healing analyses (c); MMP-2 and MMP-9 levels were tested utilizing western blot (d). *P < 0.05 vs. NC exosome + mimics NC group, ^#^P < 0.05 vs. FOXD2-AS1 exosome + mimics NC group.

The results of CCK-8 and EdU assays demonstrated that in the comparison with NC exosome + mimics NC group, the proliferation of HaCaT cells was notably reduced in NC exosome + miR-185-5p mimics group, but elevated in FOXD2-AS1 exosome + mimics NC group (Figure 5a and 5b). Meanwhile, the proliferation of HaCaT cells in FOXD2-AS1 exosome + miR-185-5p mimics group was elevated relative to NC exosome + miR-185-5p mimics group, but was reduced relative to FOXD2-AS1 exosome + mimics NC group (Figure 5a and 5b). Moreover, the results of Figure 4c showed that the migration of HaCaT cells was notably decreased in NC exosome + miR-185-5p mimics group relative to NC exosome + mimics NC group, but increased in FOXD2-AS1 exosome + mimics NC group. The migration of HaCaT cells in FOXD2-AS1 exosome + miR-185-5p mimics group was increased compared with NC exosome + miR-185-5p mimics group but was decreased relative to FOXD2-AS1 exosome + mimics NC group (Figure 5c). Similarly, the results are also demonstrated using western blot. MMP-2 and MMP-9 level was lower in NC exosome + miR-185-5p mimics group relative to NC exosome + mimics NC group, but higher in FOXD2-AS1 exosome + mimics NC group (Figure 5d). Meanwhile, the expressions of MMP-2 and MMP-9 level in FOXD2-AS1 exosome + miR-185-5p mimics group was elevated relative to NC exosome + miR-185-5p mimics group and down-regulated relative to FOXD2-AS1 exosome + mimics NC group (Figure 5d).

### miR-185-5p negatively targeted ROCK2

**Figure 6.**
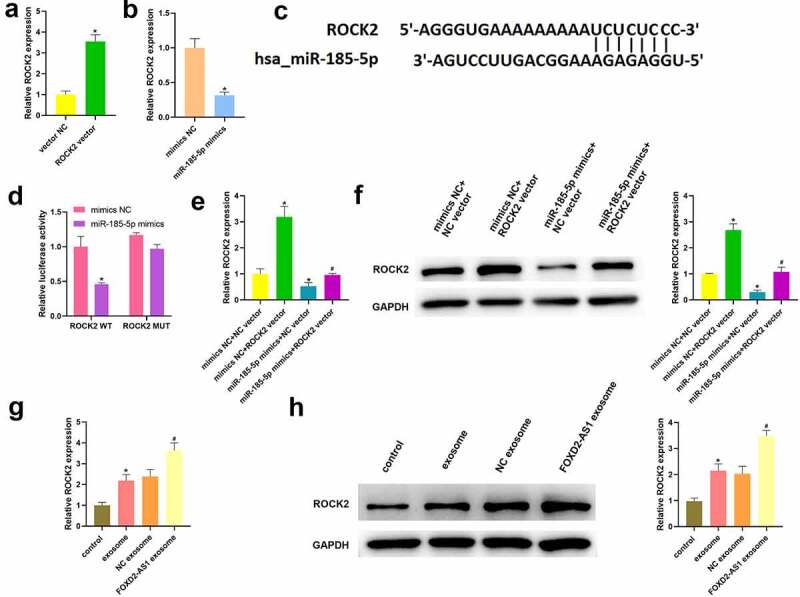
miR-185-5p negatively targeted ROCK2. (a) After transfection of ROCK2 overexpression vector, the expression of ROCK2 was detected using qRT-PCR. (b) Following transfection of miR-185-5p mimics, ROCK2 level was detected using qRT-PCR. (c) The bind sites of miR-185-5p and ROCK2. (d) A dual luciferase reporter assay verified the binding relationship between miR-185-5p and ROCK2. After co-transfection with ROCK2 overexpression vector and miR-185-5p mimics, ROCK2 expressions were analysed using qRT-PCR (e) and western blot (f). *P < 0.05 vs. vector NC, mimics NC, mimics NC +ROCK2 WT, and mimics NC+ NC vector group, ^#^P < 0.05 vs miR-185-5p mimics + NC vector and NC exosome group.

As shown in Figure 6a, the expression of ROCK2 was upregulated in HaCaT cell followed by transfection of ROCK2 overexpression vector. Additionally, the expression of ROCK2 was downregulated followed by transfection of miR-185-5p mimics (Figure 6b). Figure 6c is predicted for binding sites between miR-185-5p and ROCK2. Figure 6d proves miR-185-5p mimics decreased luciferase activity in the ROCK2-WT group, while had no function on luciferase activities in ROCK2-MUT group. Furthermore, Figure 5e and 5f data showed that ROCK2 expression were higher in mimics NC + ROCK2 vector group than that in mimics NC + NC vector group, but lower in miR-185-5p mimics + NC vector group. Meanwhile, mRNA and protein expression of ROCK2 in miR-185-5p mimics + ROCK2 vector group was reduced relative to mimics NC + ROCK2 vector group, but higher than that in miR-185-5p mimics + NC vector group (Figure 6e and 6f). The above data suggested that miR-185-5p could negatively target ROCK2. Moreover, we also found that exosomes treatment significantly induced ROCK2 expression, and lncRNA FOXD2-AS1 overexpression-transfected exosomes further promoted the expression of ROCK2 (Figure 6g and 6h).

### miR-185-5p overexpression inhibited HaCaT cell migration and proliferation through down-regulating ROCK2

**Figure 7.**
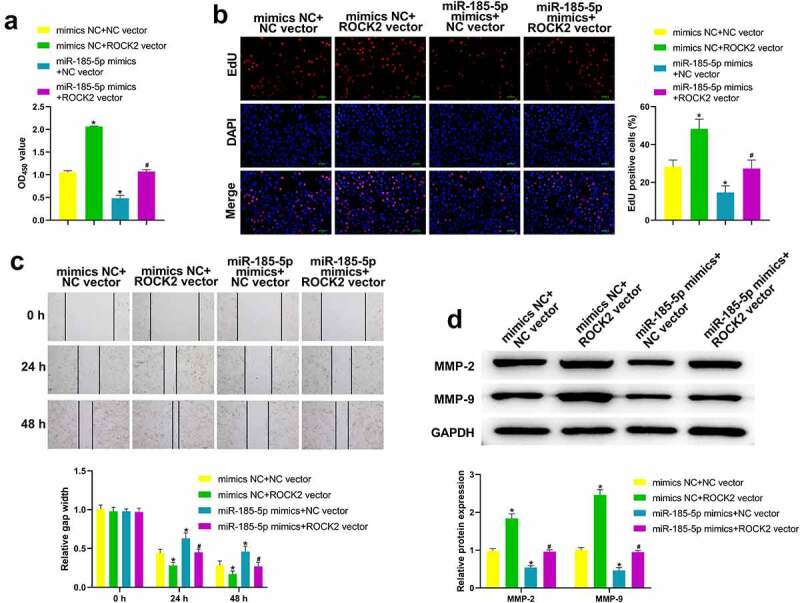
miR-185-5p overexpression inhibited the migration and proliferation of HaCaT cells through down-regulating ROCK2. Following different treatment, the HaCaT cell variety was evaluated utilizing CCK-8 analyses (a); HaCaT cell proliferation was analysed with EdU analyses (b); HaCaT cell migration at 0, 24, 48 h was analysed with wound healing analyses (c); the expressions of MMP-2 and MMP-9 levels were tested utilizing western blot (d). *P < 0.05 vs mimics NC +NC vector group, ^#^P < 0.05 vs. miR-185-5p mimics + NC vector group.

As shown in Figure 7a and 7b, the proliferation of HaCaT cells was notably elevated in mimics NC + ROCK2 vector group than that in mimics NC + NC vector group, but downregulated in miR-185-5p mimics + NC vector group. HaCaT cell proliferation in miR-185-5p mimics + ROCK2 vector group was decreased compared with mimics NC + ROCK2 vector group but was increased compared with miR-185-5p mimics + NC vector group (Figure 7a and 7b). In comparison with mimics NC + NC vector group, HaCaT cell migration was promoted in mimics NC + ROCK2 vector group and decreased in miR-185-5p mimics + NC vector group (Figure 7c). Meanwhile, HaCaT cell migration in miR-185-5p mimics + ROCK2 vector group was inhibited relative to mimics NC + ROCK2 vector group, but was elevated relative to miR-185-5p mimics + NC vector group (Figure 7c). Moreover, western blot results confirmed that MMP-2 and MMP-9 level was higher in mimics NC + ROCK2 vector group than those in mimics NC + NC vector group, but lower in miR-185-5p mimics + NC vector group (Figure 7d). Meanwhile, MMP-2 and MMP-9 level in miR-185-5p mimics + ROCK2 vector group was decreased when compared with mimics NC + ROCK2 vector group, and increased in miR-185-5p mimics + NC vector group (Figure 7d).

## Discussion

A basic characteristic of wound healing is the repair of the intact epidermal barrier via reepithelialization [22]. It is reported that keratinocytes proliferation and migration are closely related to reepithelialization and closure of the wound gap [23,24]. In the current study, highly expressed lncRNA FOXD2-AS1 in ADMSCs derived exosomes promoted HaCaT cell migration and proliferation via modulating the miR-185-5p/ROCK2 axis.

Currently, increasing evidences have suggested that the novel role of exosomes as cell communication bodies is dependent on exosomal cargo, including lncRNAs, microRNAs (miRNAs) and proteins [25,26]. Furthermore, accumulating evidences relate regulatory lncRNAs to human diseases. LncRNA FOXD2-AS1 may accelerate proliferation and migration activities in a various of cells [20,27,28]. Interestingly, a study has proven that exosomal lncRNA FOXD2-AS1 can also act as the promising biomarkers for the diagnostics of colorectal cancer [14]. Therefore, we explored the effects of exosomal lncRNA FOXD2-AS1 derived from ADMSCs in wound healing. Our data demonstrate that highly expressed lncRNA FOXD2-AS1 in ADMSCs-exosomes might accelerate HaCaT cell migration and proliferation.

Recent research data suggest that miRNAs are promising tools for the treatment and diagnosis of skin wound healing because miRNAs are important regulators of cellular physiology and pathology [29]. miR-200b/c-3p can modulate epithelial plasticity and repress cutaneous wound healing through the regulation of TGF-β-mediated RAC1 pathway [30]. It is interesting that lncRNA interacts with miRNA and the interactions play critical roles in determining cell fate [31]. Adipose-derived stem cells-exosomes containing lncRNA MALAT1 can promote wound healing via targeting miR-124 [25]. LncRNA GAS5 is proved to accelerate diabetic wound healing and promote lymphangiogenesis by miR-217/Prox1 axis [32]. Through bioinformatics analyses and Dual-luciferase reporter assay, we verified that lncRNA FOXD2-AS1 targetly regulates miR-185-5p. In addition, lncRNA FOXD2-AS1 directly interacts with miR-185-5p as miRNA sponge and promotes tumour progression, including colorectal cancer [33], papillary thyroid cancer [34], and glioma [35]. In the current study, lncRNA FOXD2-AS1 overexpression in ADMSCs-exosomes promoted the migration and proliferation of HaCaT cells via down-regulating miR-185-5p.

MiRNAs are verified to reverse gene expression via degrading mRNA and inhibiting translation [36]. ROCK2 is widely involved in cell biological activities and plays an important role in controlling various cell phenomena [37]. ROCK2 has been suggested to negatively regulate the Parkin-dependent mitophagy pathway [38]. ROCK2 contributes to diabetes-induced impaired cardiac Ca^2+^ homoeostasis [39]. ROCK2-induced glycolysis and proliferation in osteosarcoma [40]. ROCK2 promotes osteosarcoma growth and metastasis by modifying the PFKFB3 ubiquitination and degradation [37]. ROCK2 promotes invasion and metastasis in hepatocellular carcinoma through disturbing MKP1 [41]. Croze et al. have reported that the suppression of ROCK can promote attachment, proliferation, and wound closure in human embryonic stem cell-derived retinal pigmented epithelium [42]. Additionally, miR-203 overexpression inhibits the proliferation of epidermal stem cells through the downregulation of ROCK2 associated with Notch and Wnt pathways, leading to a delayed wound healing [43]. We verified miR-185-5p can targetly regulate ROCK2 in this study. In addition, Niu et al. have proved that miR-185-5p may inhibit hepatocellular carcinoma cell migration through targeting ROCK2 [44]. In the present study, we demonstrated that miR-185-5p overexpression could inhibit the migration and proliferation of HaCaT cells through down-regulating ROCK2.

## Conclusion

In the current study, highly expressed lncRNA FOXD2-AS1 in ADMSCs derived exosomes might accelerate HaCaT cell migration and proliferation via modulating the miR-185-5p/ROCK2 axis, revealing that exosomal lncRNA FOXD2-AS1 derived from ADMSCs may be a novel therapeutic strategy for wound healing.

## Materials and Methods

### Isolation and identification of human ADMSCs

The adipose tissues were acquired from the discarded tissue of a 5-year-old female patient who underwent full-layer abdominal skin transplant surgery in our hospital. The protocol of this research has been approved by the Ethics Committee of Weifang Medical University. All patients have signed written informed consent. First of all, adipose tissues were cut into cubes of about 1 mm^3^, which were free of fibre or blood vesicles. Following washed using 0.9% NS, adipose tissues were digested applying 0.75% type I collagenase at 37°C for 1 h. Subsequently, the resuspended cells were transplanted into DMEM medium (Gibco, USA). The 3^rd^ ADMSCs were used for the subsequent experiment. ADMSCs characterization was evaluated utilizing flow cytometry, as described previously [45]. The antibodies for CD44 (1:100; #ab243894), CD105 (1:200; #ab231774), CD31 (1:200; #ab9498), and HLA-DR (1:100; #ab20181) were obtained from Abcam.

#### LncRNA FOXD2-AS1 transfection

The lncRNA FOXD2-AS1 overexpression plasmid, si- FOXD2-AS1, and negative control (FulenGen, Guangzhou, China) were incubated with ADMSCs cells for 48 h by Lipofectamine 3000 (Thermo Fisher Scientific, USA).

### Isolation and identification of exosomes

At 48  h posttransfection, exosome was collected applying the Ribo™ Exosome Isolation Reagent (Ribobio, Guangzhou, China). The extracted exosome morphology was determined using transmission electron microscopy (TEM; Leica, Germany). Exosome size distribution was analysed using the nanoparticle tracking analysis (NTA) as previously described [46]. Finally, the expression of CD9, CD63 and TSG101 was evaluated by western blotting.

### Cell culture and treatment

Human keratinocyte cells (HaCaT cells) were supplied by NanJing Cobioer Biosciences Co., Ltd (China). Cells were cultured in a DMEM medium containing 10% FBS and 100 U/ml penicillin and streptomycin at 37°C with 5% CO_2_. HaCaT cells (1 × 10^6^) were seeded into a 100 mm dish and incubated with 20 μg exosomes isolated from the ADMSCs for 24 h, then the culture supernatants were collected for the subsequent experiment. The lncRNA FOXD2-AS1 overexpression plasmid (FOXD2-AS1 overexpression), miR-185-5p mimics, ROCK2 overexpression plasmid (ROCK2 vector) and their corresponding negative controls were incubated with HaCaT cells for 48 h by Lipofectamine 3000 (Thermo Fisher Scientific, USA). The FOXD2-AS1 overexpression plasmid and ROCK2 overexpression plasmid were supplied by FulenGen (Guangzhou, China). AndmiR-185-5p mimic was obtained from GenePharma (Shanghai, China).

### Cell counting kit-8 (CCK-8) assay

The cell proliferation was evaluated by a CCK-8 Cell Proliferation and Cytotoxicity Assay Kit (DOJINDO, Japan). HaCaT cells (1 × 10^4^ cells/well) were cultured in a 96-well plate. CCK-8 solution (10 μL) was added into plate, subsequently the cells were further incubated for 48 h at 37°C with 5% CO_2_. Optical density at 450 nm was analysed applying a microplate reader.

### 5-ethynyl-20-deoxyuridine (EdU) assay

The rates of the proliferating cells were determined with an EdU proliferation kit (Beyotime, Shanghai, China). HaCaT cells (1 × 10^4^ cells/well) were incubated in a 96-well plate, and the medium with EdU solution (50 μM) was added. Following 120 min of culture, the 4′, 6-diamidino-2-phenylindole (DAPI, Beyotime) was added into each well of the plates and shielded from light for 30 min. Finally, the EdU-positive cells were visualized using a fluorescent microscope and quantified applying ImageJ software.

### Wound healing assay

HaCaT cells (4 × 10^4^ cells/well) were plated into a 96-well plate and cultured till reaching 90% confluence. After that, sterile pipette tips were applied to scrape cell confluent monolayer in a standardized manner, creating a cell-free zone in each well. After the culture of HaCaT cells for 48 h at 37°C, the wound’s distance at 0, 24, and 48 h was photographed under the light microscope.

### Dual luciferase reporter assay

Bioinformatics website StarBase (http://starbase.sysu.edu.cn/index.php) and TargetScanHuman (http://www.targetscan.org/vert_72/) were used to identify potential binding sites for lncRNA FOXD2-AS1, miR-185-5p, and ROCK2. ROCK2 3’-UTRs including wild-type (WT) and mutant (MUT) miR-185-5p binding site or FOXD2-AS1 3’-UTRs including WT and MUT miR-185-5p binding site were inserted into pmir-reporter vector to construct ROCK2-WT and ROCK2-MUT reporter vectors or FOXD2-AS1-WT and FOXD2-AS1-MUT reporter vectors. After that, HaCaT cells transfected these reporter vectors, miR-185-5p mimics and mimics NC for 2 d utilizing Lipofectamine 2000 (Thermo Fisher Scientific, USA). Luciferase activity was determined utilizing Dual-Luciferase Reporter Kit (Vazyme, Nanjing, China).

### qRT-PCR

Total RNA was extracted with TRIzol (Beyotime, Beijing, China). The complementary DNA was synthesized using the *EasyScript*® First-Strand cDNA Synthesis SuperMix (Transgen, Beijing, China) and *TransScript*® miRNA First-Strand cDNA Synthesis SuperMix (Transgen) based on the instructions of the manufacturer. Afterwards, RT-PCR was carried out with the *TransScript*®Green Two-Step qRT-PCR SuperMix (Transgen) or *TransScript*® Green miRNA Two-Step qRT-PCR SuperMix (Transgen). The primer sequences were as follows: FOXD2-AS1-sence: 5′-TGGACCTAGCTGCAGCTCCA-3′, antisense: 5′-AGTTGAAGGTGCACACACTG-3′; miR-185-5p-sense: 5′-GCGGCGGTGGAGAGAAAGGCAG-3′, antisense: 5′-ATCCAGTGCAGGGTCCGAGG-3′; ROCK2-sence: 5′-AACGTCAGGATGCAGATGGG-3′, antisense: 5′-CAGCCAAAGAGTCCCGTTCA-3′; GAPDH-sence: 5′-GTTGCAACCGGGAAGGAAAT-3′, antisense: 5′-GCCCAATACGACCAAATCAGA-3′ and U6-sence: 5′-CAGCACATATACTAAAATTGGAACG-3′, antisence: 5′-ACGAATTTGCGTGTCATCC-3′.

### Western blot

Total protein was extracted by Radio Immunoprecipitation Assay buffer (Thermo Fisher Scientific, USA). Equal amounts of protein (50 μg per lane) were detached by 10% SDS-PAGE and transferred onto nitrocellulose membranes. After that, the primary antibodies (CD9, #ab236630; CD63, #ab134045; TSG101, #ab125011; MMP-2, #ab92536; MMP-9, #ab137867; ROCK2, #ab125025; GAPDH, #ab181602, Abcam, UK) were conducted with membranes overnight at 4°C. Subsequently, the secondary antibody was utilized to incubate membranes for 1 h. The blots were detected using enhanced chemiluminescence (Thermo Fisher Scientific, USA).

### Statistical analysis

Data obtained in the study were represented as the mean ± SD of three independent experimental repeats and measured utilizing GraphPad Prism 8.0. Statistical differences were determined by Student's t-test or one-way ANOVA. A p-value <0.05 was considered to be significant in all experiments.

## Data Availability

The datasets used and analysed during the current study are available from the corresponding author on reasonable request.

## List of abbreviations

Adipose mesenchymal stem cells (ADMSCs), Mesenchymal stem cells (MSCs)

## References

[1] Du H, Zhou Y, Suo Y, et al. CCN1 accelerates re-epithelialization by promoting keratinocyte migration and proliferation during cutaneous wound healing. Biochem Biophys Res Commun. 2018;505(4):966–13. 30361094 10.1016/j.bbrc.2018.09.001

[2] Pradhan L, Andersen ND, Nabzdyk C. Wound-healing Abnormalities in Diabetes and New Therapeutic Interventions. *US Endocrinol*. 2007;5:68.

[3] Eming SA, Martin P, Tomic-Canic M. Wound repair and regeneration: mechanisms, signaling, and translation. Sci Transl Med. 2014;6:265sr6–265sr6. 25473038 10.1126/scitranslmed.3009337 PMC4973620

[4] Vizoso FJ, Eiro N, Costa L, et al. Mesenchymal Stem Cells in Homeostasis and Systemic Diseases: hypothesis, Evidences, and Therapeutic Opportunities. Int J Mol Sci. 2019;20:3738. 31370159 10.3390/ijms20153738 PMC6696100

[5] Lee MJ, Kim JK, Kim MY, et al. Proteomic Analysis of Tumor Necrosis Factor-α-Induced Secretome of Human Adipose Tissue-Derived Mesenchymal Stem Cells. J Proteome Res. 2010;9(4):1754–1762. 20184379 10.1021/pr900898n

[6] Liu W, Sui X, Liu Z, et al. Exosomal lncRNA-p21 derived from mesenchymal stem cells protects epithelial cells during LPS-induced acute lung injury by sponging miR-181. Acta Biochim Biophys Sin (Shanghai). 2021;53:748–757. 33891698 10.1093/abbs/gmab043

[7] Tian X, Bo J, Hong Y. Effect of adipose-derived mesenchymal stem cell exosomes on the proliferation and migration of keratinocytes and the underlying mechanisms. Chin J Tissue Eng Res. 2019;23:68.

[8] Ma T, Fu B, Yang X, et al. Adipose mesenchymal stem cell‐derived exosomes promote cell proliferation, migration, and inhibit cell apoptosis via Wnt/β‐catenin signaling in cutaneous wound healing. J Cell Biochem. 2019;123:10847. 10.1002/jcb.28376 30681184

[9] Zhang Y, Han F, Gu L, et al. Adipose mesenchymal stem cell exosomes promote wound healing through accelerated keratinocyte migration and proliferation by activating the AKT/HIF-1α axis. J Mol Histol. 2020;51:375. 32556903 10.1007/s10735-020-09887-4

[10] Heo JS, Kim S, Yang CE, et al. Human Adipose Mesenchymal Stem Cell-Derived Exosomes: a Key Player in Wound Healing. Tissue Engineering Regenerat Medi. 2021;18(4):537–548. 10.1007/s13770-020-00316-x PMC8325736 33547566

[11] Li H, Wang J, Xin Z, et al. Exosomes derived from human adipose mesenchymal stem cells accelerates cutaneous wound healing via optimizing the characteristics of fibroblasts. Rep. 2016;6:32993. 10.1038/srep32993 PMC5018733 27615560

[12] Gezer U, Zgür E, Cetinkaya M, et al. Long non-coding RNAs with low expression levels in cells are enriched in secreted exosomes. Cell Biol Int. 2014;38:1076–1079. 24798520 10.1002/cbin.10301

[13] Qian L, Pi L, Fang BR, et al. Adipose mesenchymal stem cell-derived exosomes accelerate skin wound healing via the lncRNA H19/miR-19b/SOX9 axis, Laboratory Investigation. 10.1038/s41374-021-00611-8 34045678

[14] Yu M, Song XG, Zhao YJ, et al. Circulating Serum Exosomal Long Non-Coding RNAs FOXD2-AS1, NRIR, and XLOC_009459 as Diagnostic Biomarkers for Colorectal Cancer. Front Oncol. 2021;11:618967. 33777763 10.3389/fonc.2021.618967 PMC7996089

[15] Chen Z, Zhang Z, Zhao D, et al. Long Noncoding RNA (lncRNA) FOXD2-AS1 Promotes Cell Proliferation and Metastasis in Hepatocellular Carcinoma by Regulating MiR-185/AKT Axis. Med Sci Monit. 2019;25:9618–9629. 31841454 10.12659/MSM.918230 PMC6929557

[16] Gao J, Liu F, Zhao X, et al. Long non-coding RNA FOXD2-AS1 promotes proliferation, migration and invasion of ovarian cancer cells via regulating the expression of miR-4492. Exp Ther Med. 2021;21(4):307. 33717250 10.3892/etm.2021.9738 PMC7885078

[17] Chen G, Sun W, Hua X, et al. Long non-coding RNA FOXD2-AS1 aggravates nasopharyngeal carcinoma carcinogenesis by modulating miR-363-5p/S100A1 pathway. Gene. 2018;645:76–84. 29248577 10.1016/j.gene.2017.12.026

[18] Zhang L, Bo H, Chen T, et al. FOXD2-AS1 promotes migration and invasion of head and neck squamous cell carcinoma and predicts poor prognosis. Future Oncology (London, England). 2020;16(28):2209–2218. 32762453 10.2217/fon-2020-0410

[19] Zhang Y, Chen X. lncRNA FOXD2-AS1 affects trophoblast cell proliferation, invasion and migration through targeting miRNA. Zygote. 2020;1–8. DOI:10.1017/S0967199419000807 31928563

[20] Zhao Q, Zhao F, Liu C, et al. LncRNA FOXD2-AS1 promotes cell proliferation and invasion of fibroblast-like synoviocytes by regulation of miR-331-3p/PIAS3 pathway in rheumatoid arthritis. Autoimmunity. 2021;54:254–263. 34030529 10.1080/08916934.2021.1919879

[21] Wang Y, Cao L, Wang Q, et al. LncRNA FOXD2-AS1 induces chondrocyte proliferation through sponging miR-27a-3p in osteoarthritis. Artif Cells Nanomed Biotechnol. 2019;47:1241–1247. 30945573 10.1080/21691401.2019.1596940

[22] Seeger MA, Paller AS. The Roles of Growth Factors in Keratinocyte Migration. Adv Wound Care. 2015;4(4):213. 10.1089/wound.2014.0540 PMC4397993 25945284

[23] Gurtner GC, Werner S, Barrandon Y, et al. Wound Repair and Regeneration. Nature. 2008;453(7193):314–321. 18480812 10.1038/nature07039

[24] Eric T, Ma Q, Castillo DE, et al. The Effects of Aloe vera on Wound Healing in Cell Proliferation, Migration, and Viability. *Wound Compend Clin Res Pract*. 2018;30:263 30256753

[25] He L, Zhu C, Jia J, et al. ADSC-Exos containing MALAT1 promotes wound healing by targeting miR-124 through activating Wnt/β-catenin pathway. Biosci Rep. 2020;40(5). DOI:10.1042/BSR20192549 PMC7214401 32342982

[26] Chen C, Luo Y, He W, et al. Exosomal long noncoding RNA LNMAT2 promotes lymphatic metastasis in bladder cancer. J Clin Invest. 2019;130:404–421. 10.1172/JCI130892 PMC6934220 31593555

[27] Zhao T, Zhang J, Ye C, et al. lncRNA FOXD2-AS1 promotes hemangioma progression through the miR-324-3p/PDRG1 pathway. Cancer Cell Int. 2020;20(1). DOI:10.1186/s12935-020-01277-w PMC7247140 32489325

[28] Lei C, Yang W, Wang Q, et al. LncRNA FOXD2-AS1 regulates chondrocyte proliferation in osteoarthritis by acting as a sponge of miR-206 to modulate CCND1 expression. Biomed Pharmacothe. 2018;106:1220–1226. 10.1016/j.biopha.2018.07.048 30119190

[29] Meng Z, Zhou, D, Gao, Y, et al. miRNA delivery for skin wound healing. Adv Drug Deliv Rev. 2018;129:308-318. 10.1016/j.addr.2017.12.011 29273517

[30] Tang H, Wang X, Zhang M, et al. MicroRNA-200b/c-3p regulate epithelial plasticity and inhibit cutaneous wound healing by modulating TGF-β-mediated RAC1 signaling. Cell Death Dis. 2020;11:931. 33122632 10.1038/s41419-020-03132-2 PMC7596237

[31] Cao MX, Jiang YP, Tang YL, et al. The crosstalk between lncRNA and microRNA in cancer metastasis: orchestrating the epithelial-mesenchymal plasticity. Oncotarget. 2017;8:55. 10.18632/oncotarget.13957 PMC5355358 27992370

[32] He Z.Y, Huang M, Cui X, et al. Long noncoding RNA GAS5 accelerates diabetic wound healing and promotes lymphangiogenesis via miR-217/Prox1 axis. *Mol Cell Endocrinol*. 2021;532:111283. 33865922 10.1016/j.mce.2021.111283

[33] Zhu Y, Qiao L, Zhou Y, et al. Long non-coding RNA FOXD2-AS1 contributes to colorectal cancer proliferation through its interaction with microRNA-185-5p. Cancer Sci. 2018;109:2235–2242. 29737580 10.1111/cas.13632 PMC6029818

[34] Li H, Han Q, Chen Y, et al. Upregulation of the long non-coding RNA FOXD2-AS1 is correlated with tumor progression and metastasis in papillary thyroid cancer. Am J Transl Res. 2019;11:5457–5471. 31632522 PMC6789238

[35] Ni W, Xia Y, Bi Y, et al. FoxD2-AS1 promotes glioma progression by regulating miR-185-5P/HMGA2 axis and PI3K/AKT signaling pathway. Aging (Albany NY). 2019;11:1427–1439. 30860979 10.18632/aging.101843 PMC6428107

[36] Bartel DP. MicroRNAs: genomics, Biogenesis, Mechanism, and Function. Cell. 2004;116(2):281–297. 14744438 10.1016/s0092-8674(04)00045-5

[37] Deng X, Yi X, Deng J, et al. ROCK2 promotes osteosarcoma growth and metastasis by modifying PFKFB3 ubiquitination and degradation. Exp Cell Res. 2019;385:111689. 31678169 10.1016/j.yexcr.2019.111689

[38] Mani S, Jindal D, Chopra H, et al. ROCK2 inhibition: A futuristic approach for the management of Alzheimer's disease. *Neurosci Biobehav Rev*. 2022;142:104871. 36122738 10.1016/j.neubiorev.2022.104871

[39] Soliman H, Nyamandi V, Garcia-Patino M, et al. ROCK2 promotes ryanodine receptor phosphorylation and arrhythmic calcium release in diabetic cardiomyocytes. Int J Cardiol. 2019;281:90–98. 30728103 10.1016/j.ijcard.2019.01.075

[40] Deng B, Deng J, Yi, X. ROCK2 Promotes Osteosarcoma Growth and Glycolysis by Up-Regulating HKII via Phospho-PI3K/AKT Signalling. Cancer Manag Res. 2021. 10.2147/CMAR.S279496 PMC7823140 33500659

[41] Du Y, Lu S, Ge J, et al. ROCK2 disturbs MKP1 expression to promote invasion and metastasis in hepatocellular carcinoma. Am J Cancer Res. 2020;10:884–896. 32266097 PMC7136912

[42] Croze RH, Thi W, J.Clegg DO. ROCK Inhibition Promotes Attachment, Proliferation, and Wound Closure in Human Embryonic Stem Cell–Derived Retinal Pigmented Epithelium. Trans Vision Sci Technol. 2016;5(6):7. 10.1167/tvst.5.6.7 PMC5132148 27917311

[43] Liu J, Shu B, Zhou Z, et al. Involvement of miRNA203 in the proliferation of epidermal stem cells during the process of DM chronic wound healing through Wnt signal pathways. Stem Cell Res Ther. 2020;11. DOI:10.1186/s13287-020-01829-x PMC7422611 32787903

[44] Niu Y, Tang G. miR1855p targets ROCK2 and inhibits cell migration and invasion of hepatocellular carcinoma. Oncol Lett. 2019. DOI:10.3892/ol.2019.10144 PMC6507519 31105794

[45] Dai XW, Luo W, Lv CL. lncRNAMIAT facilitates the differentiation of adipose derived mesenchymal stem cells into lymphatic endothelial cells via the miR495/Prox1 axis. Mol Med Rep. 2021;23. DOI:10.3892/mmr.2021.11962 33760182

[46] Liu H, Sun X, Gong X, et al. Human umbilical cord mesenchymal stem cells derived exosomes exert antiapoptosis effect via activating PI3K/Akt/mTOR pathway on H9C2 cells. Journal of Cellular Biochemistry. 2019;120:14455–14464. 30989714 10.1002/jcb.28705

